# Supplementary material for: Authentication of Argan (Argania spinosa L.) Oil Using Novel DNA-Based Approaches: Detection of Olive and Soybean Oils as Potential Adulterants
Source: Foods. 2022 Aug 18;11(16):2498. doi: 10.3390/foods11162498 (PMC9407626; doi:10.3390/foods11162498)
Supplement: Supplementary file 1 [file foods-11-02498-s001.zip › foods-1837924-supplementary.pdf]

Supplementary material

Table S1. Results of PCR amplification targeting the ITS2, *matK* and *lectin* genes of argan, olive and soybean, respectively, and a universal eukaryotic DNA region of several relevant plant species for cross-reactivity testing.

Common name	Plant species	Qualitative PCR			
		18S rRNA (EG-F/R)	ITS2 (ITS2A-F/R)	*matK* (matKO-F/R)	Lectin (LE1/LE2)
Argan	*Argania spinosa*	+	+	-	-
Olive	*Olea europaea*	+	-	+	-
Soybean	*Glycine max*	+	-	-	+
Sunflower	*Helianthus annuus*	+	-	-	-
Rapeseed	*Brassica napus*	+	-	-	-
Maize	*Zea mays*	+	-	-	-
Almond	*Prunus dulcis*	+	-	-	-
Brazil nut	*Bertholletia excelsa*	+	-	-	-
Cashew nut	*Anacardium occidentale*	+	-	-	-
Hazelnut	*Corylus avellana*	+	-	-	-
Macadamia nut	*Macadamia tetraphylla*	+	-	-	-
Pine nut	*Pinus pinea*	+	-	-	-
Pistachio	*Pistacia vera*	+	-	-	-
Walnut	*Juglans regia*	+	-	-	-
Peanut	*Arachis hypogaea*	+	-	-	-
Oat	*Avena sativa*	+	-	-	-
Rye	*Secale cereale*	+	-	-	-

Table S2. Commercial argan oil samples tested PCR amplification targeting the ITS2, *matK* and *lectin* genes of argan, olive and soybean, respectively.

Sample	Application	Label	Qualitative PCR		
			ITS2 (ITS2A-F/R)	*matK* (matKO-F/R)	Lectin (LE1/LE2)
S1	Cosmetic	Huile d’Argane (non-torrifeée) (Produit Biologique, Marocco)	+	-	-
S2	Cosmetic	Huile d’Argane (Visage, corps et cheveux) (Bio, Marocco)	+	-	-
S3	Cosmetic	Huile d’Argane, beauté (100% naturel, Bio, Marocco)	+	-	-
S4	Food	Huile d’Argane Alimentaire (Produit Biologique, IGP, Marocco)	+	-	-
S5	Food	Huile Vierge Argan Alimentaire (Torréfié Pressée à Froid, Bio, Marocoo)	+	-	-

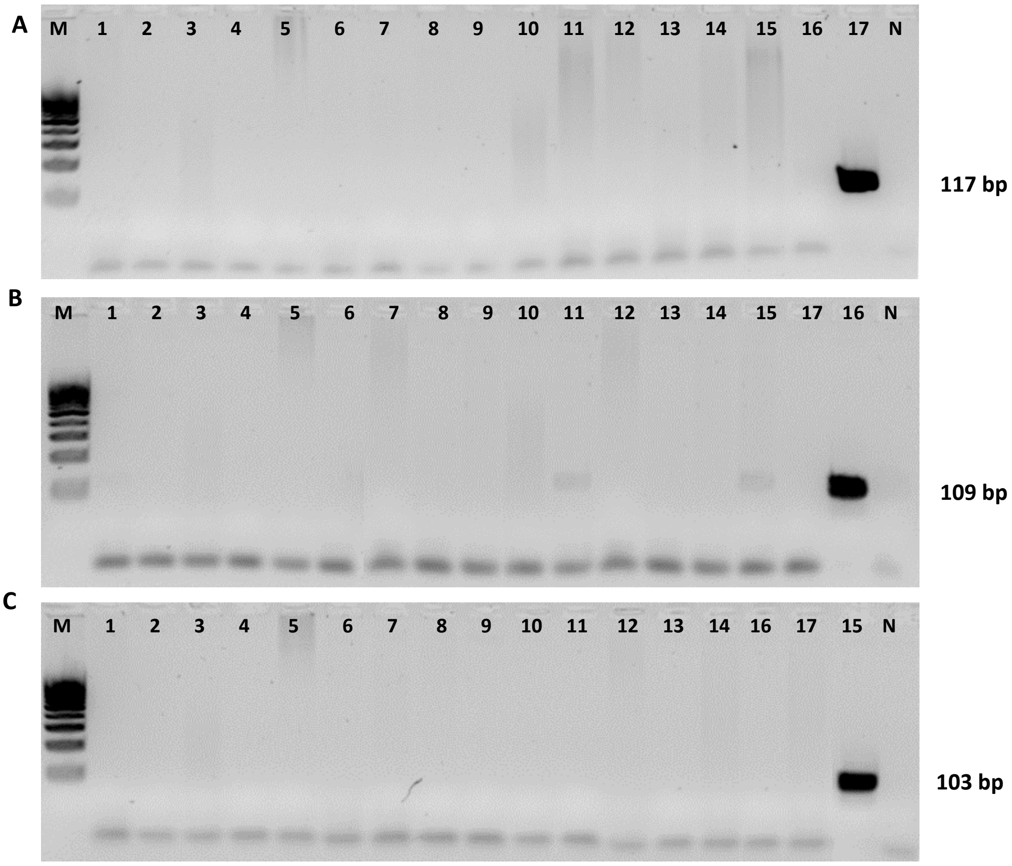

Figure S1. Agarose gel electrophoresis of PCR products targeting ITS, *matK* and *lectin* genes of *A. spinosa* (A), *O. europaea* (B) and *G. max* (C), respectively, for cross-reactivity assessment. Legend: Lane M, 100 bp molecular marker (Bioron, Ludwigshafen, Germany); lane 1, macadamia nut; lane 2, cashew nut; lane 3, peanut; lane 4, almond; lane 5, walnut, lane 6, hazelnut; lane 7, Brazil nut; lane 8, pine nut; lane 9, pistachio nut; lane 10, rapeseed; lane 11, sunflower; lane 12, oat; lane 13, rye; lane 14, maize; lane 15, soybean; lane 16, olive; lane 17, argan; lane N, negative control.

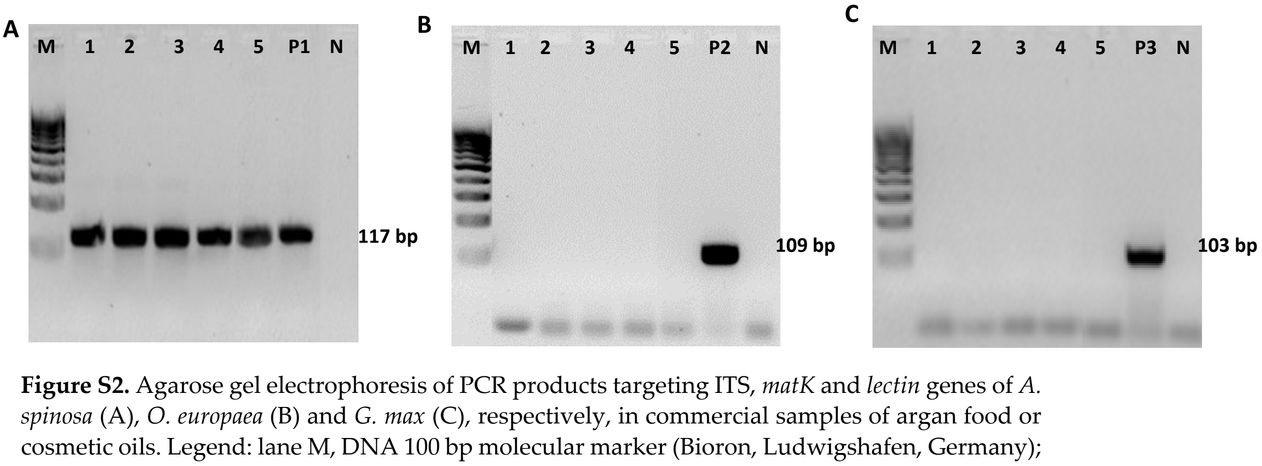

Figure S2. Agarose gel electrophoresis of PCR products targeting ITS, *matK* and *lectin* genes of *A. spinosa* (A), *O. europaea* (B) and *G. max* (C), respectively, in commercial samples of argan food or cosmetic oils. Legend: lane M, DNA 100 bp molecular marker (Bioron, Ludwigshafen, Germany); lanes 1-3, argan oil cosmetic grade S1, S2 and S3, respectively; lanes 4 and 5, argan oil food grade S4 and S5, respectively; lane P1, positive control for argan; lane P2, positive control for olive; lane P3, positive control for soybean; lane N, negative control.
